# Numerical Simulation of Layered Bimetallic ZChSnSb8Cu4/Steel TIG-MIG Hybrid Welding Based on Simufact

**DOI:** 10.3390/ma16155346

**Published:** 2023-07-29

**Authors:** Hao Guo, Chenkang Fan, Shufeng Yang, Jianmei Wang, Wenle Pei, Zhibing Chu

**Affiliations:** 1 Engineering Research Center Heavy Machinery Ministry of Education, Taiyuan University of Science and Technology, Taiyuan 030024, China; guohaoustb@163.com (H.G.); fanchenkangtyust@163.com (C.F.); tyustedu2023@126.com (J.W.);; 2 School of Metallurgical and Ecological Engineering, University of Science and Technology Beijing, Beijing 100083, China; 3 State Key Laboratory of Advanced Metallurgy, University of Science and Technology Beijing, Beijing 100083, China; 4 College of Material Science and Engineering, Taiyuan University of Science and Technology, Taiyuan 030024, China

**Keywords:** layered bimetallic ZChSnSb8Cu4/steel, TIG-MIG hybrid welding, thermal-mechanical coupling simulation, double ellipsoid heat source

## Abstract

Considering the problem of the weak bonding interface structure between the rolling mill oil and film bearing bushings of Babbitt alloy and steel substrate, a numerical simulation of the layered bimetallic ZChSnSb8Cu4/steel by tungsten inert gas (TIG)-metal inert gas (MIG) hybrid welding process was carried out using Simufact Welding software (version 2020). In this study, the TIG-MIG hybrid welding process was simulated to obtain the temperature field and the stress field distributions. The residual stress and the deformation of the weldment were also analyzed using the calculated results. The results showed that the temperature gradient and the thermal stress were reduced in TIG-MIG hybrid welding compared to the conventional MIG welding preparation of layered bimetal ZChSnSb8Cu4/steel, which resulted in an improvement in the structural stability of the weldment. The temperature field and deformation of TIG-MIG hybrid welding of Babbitt alloy were studied under different controlled electrode spacings and TIG welding currents, and it was found that as electrode spacing increased, so did heat loss. Furthermore, with increased TIG welding current, compressive stress increased and tensile stress at the weld decreased, and the maximum thermal efficiency of welding was with a preheating current of 60 A.

## 1. Introduction

Oil-film bearings are widely used in steel rolling, mining, electric power, metallurgy and other key equipment due to their high rigidity, low friction coefficient and low wear rate [1,2,3]. The main material of the oil-film bearing bushes is Babbitt alloy. The Babbitt alloy is white in color and the soft matrix gives it excellent compliance, embedding and occlusion resistance in terms of microstructural properties. After grinding, the hard outer convex points and the soft inner concave substrate forms a gap between the sliding surfaces, which facilitates the flow and storage of lubricant and reduced wear [4]. However, even in well-designed high-speed shaft systems, oil-films are severely damaged when bearings are subjected to cyclically varying loads and repeated start-stop conditions. At this point, the direct contact and friction between the Babbitt alloy shaft and the rotating shaft could cause severe material wear, which could easily lead to machine downtime or even catastrophic accidents [5]. Therefore, the combination of carbon structural steel and Babbitt alloy is an efficient and economical way to improve the performance and service life of oil-film bearings [6].

In the past, centrifugal casting was the most commonly used method for joining steel/Babbitt alloy composite plates. Although the centrifugal casting technique could improve serious problems, it has been shown that this method did not fundamentally solve the problem of microstructural segregation and coarse grain produced during casting [7]. For oil-film bearings, the welding technique could be considered as a better choice [8,9].

Over the past few decades, researchers have focused mainly on the manufacturing process of Babbitt alloy. From the initial development of gravity casting in the overlay welding process, many new welding methods have been invented, of which tungsten inert gas welding (TIG) and metal inert gas welding (MIG) processes have been most widely used. Wei [10] et al. used MIG arc brazing on the surface of medium carbon steel to join SnSb8Cu4, SnSb8Cu8, and SnSb11Cu6, and their weld bond strengths were 66.42, 83.07 and 75.72 MPa, respectively. The results indicated that the bond strength of layered bimetallic Babbitt alloy/steel was dramatically affected by the different composition of welding wires. Zhou [11] et al. used TIG arc brazing to join the tin-based Babbitt alloy and Q235B steel. This research concluded that the interfacial layer thickness ranged from 10.26 to 34.27 μm and the bond strength increased dramatically from 73 to 155 MPa when the welding currents were in the range of 50 to 90 A. Research showed that MIG welding was highly productive, but during the production process, due to the jumping of the cathode spot, it was easy to generate welding spatter, which made the weld structure unstable. The TIG welding process could ensure the stability of the weld structure, but the production efficiency decreased [12]. Therefore, in recent years, welding researchers had developed a new welding process, TIG-MIG hybrid welding, with the characteristics of high production efficiency and weld structure stability [13].

In recent years, TIG-MIG hybrid welding has been studied in detail by many scholars through both numerical simulations and experiments. Chen et al. [14] derived adaptive plane and volumetric heat source models for each welding method based on the experimental visualization of the arc behavior. Then, the effect of the torch angle on the weld temperature distribution and geometry was calculated and experimentally verified. Finally, the TIG-MIG hybrid welding process was simulated and analyzed. Wu et al. [15] improved their adaptive heat source and arc pressure models based on the distribution of curved tilted TIG-MIG arcs in the flat surface, a 3D transient model was established for the heat and mass transfer within the molten pool. Finally, the quantitative relationship between welding parameters, weld beam formation and molten pool behavior was established. Kanemaru et al. [16] investigated the effect of the current balance range between the TIG and MIG arcs on arc stability and arc penetration by both experimental and numerical simulation. Ding et al. [17] used TIG-MIG hybrid welding to join AZ31B magnesium alloys and 430 ferritic stainless steels with different thicknesses of copper interlayers. However, there has been fewer investigations of the TIG-MIG hybrid welding process for layered bimetallic ZChSnSb8Cu4/steel bonding.

In the present study, the TIG-MIG hybrid welding process was performed by numerical simulation to solve the welding spatter problem that arises in the fabrication of layered bimetallic ZChSnSb8Cu4/steel by conventional MIG welding. The advantages of TIG-MIG hybrid welding compared to conventional MIG welding were investigated from the perspectives of temperature and stress filed distributions. Then, the effects of the distance between the electrodes of TIG-MIG hybrid welding and TIG welding current on the temperature and stress in the welding process were studied. Finally, the thermal efficiency of welding under different welding conditions was calculated. This study proposes an innovative application of simulation calculations of the TIG-MIG hybrid welding method applied to layered bimetallic ZChSnSb8Cu4/steel, which could provide guidance for the design of subsequent experimental TIG-MIG hybrid welding tests.

## 2. Materials and Methods

### 2.1. Analysis Process

In this paper, SolidWorks (Dassault Systèmes SolidWorks Corporation, Waltham, MA, USA), Visual Mesh (ESI, Paris, France) and JMatPro (Sente Software Ltd, Surrey Research Park, United Kingdom) were used as the pre-processing software for the numerical simulation of welding. Simufact Welding (MSC Software, Los Angeles, CA, USA) software was then used to simulate the welding process. Compared to other similar software, these software are more stable in operation, faster in calculation and better in accuracy, which can better complete the pre-processing of finite element simulation in this study.

**Figure 1:**
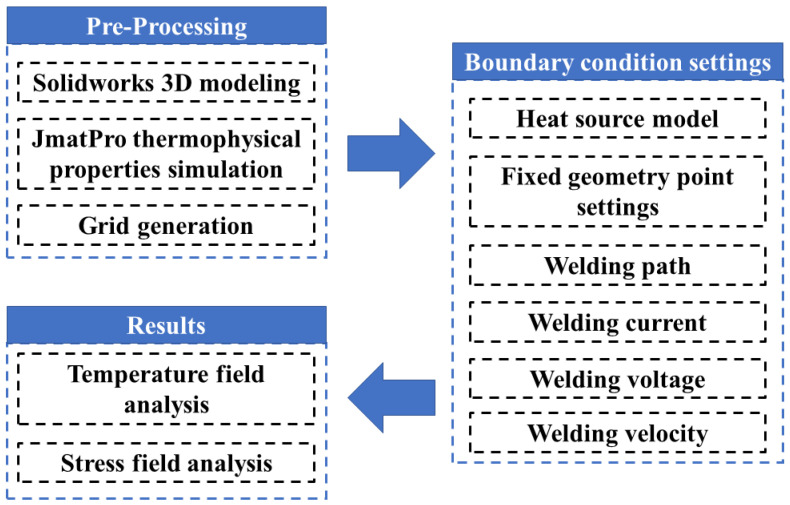
Numerical simulation flow based on Simufact Welding.

The simulation flow is shown in Figure 1. The picture shows the research plan for all the work, which involves improving the size of the geometric model and the mesh structure of the finite element simulation including the material’s thermo-physical property parameters. The finite element simulation was used to investigate the temperature field distribution and the stress field distribution law under different welding heat inputs.

### 2.2. Material Physical Property Parameters

**Figure 2:**
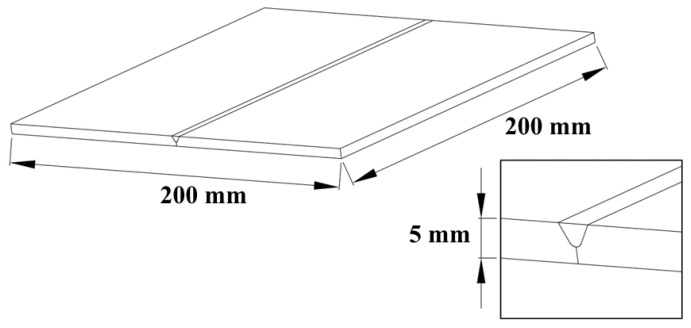
Geometry model for simulation.

In order to evaluate the results of the numerical simulation, actual experimental situations were observed. The Babbitt alloy on a Q235 flat plate with dimensions of 200 mm × 200 mm × 5 mm was selected as the simulation object, while a 60° V-shaped bevel to the flat plate was designed (China GB/T 985.1-2008). Figure 2 shows the schematic diagram of the simulation geometry model. The ambient temperature was set to 20 °C, and the other main physical thermodynamic parameters of the Q235 steel were shown in Table 1.

**Figure 3:**
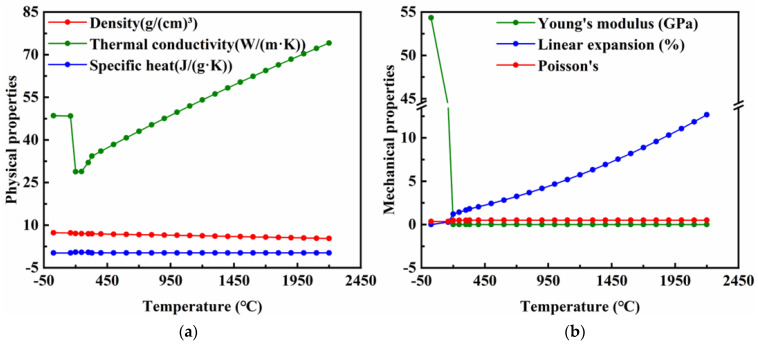
Properties of ZChSnSb8Cu4 Babbitt alloy: (**a**) Physical properties; (**b**) Mechanical properties.

The physical properties of metallic materials, such as specific heat capacity, thermal conductivity and Young’s modulus, changed dramatically as the welding temperature increased [18,19]. To calculate transient temperature fields, residual stresses and deformations with high accuracy, piecewise linear functions of the temperature values of yield stress and constant room temperature of other material properties are typically used. However, the thermophysical properties of the Babbitt alloy at high temperatures have rarely been reported. JMatPro software is widely used in welding process research due to its excellent material property simulation technology and simple operating system [20]. Table 2 and Table 3 demonstrate the main chemical compositions of the babbitt alloy and the Q235 steel, respectively. The physical property parameters of ZChSnSb8Cu4 were simulated by the JMatPro software (version 7.0), and are shown in Figure 3.

### 2.3. Mesh Structure

**Figure 4:**
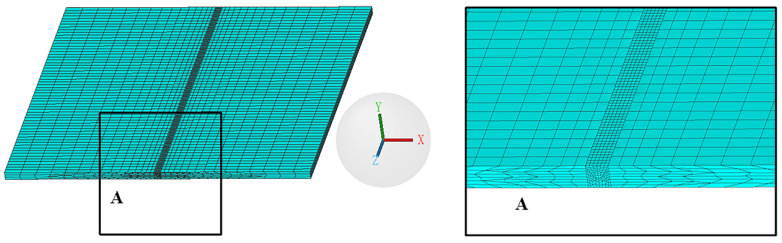
Mesh structure.

This work was modelled in SolidWorks (Dassault Systèmes SolidWorks Corporation, Waltham, MA, USA) software and then imported into Visual Mesh (ESI, Paris, France) software for mesh generation. During the actual welding process, the values of the temperature change in the weld and in the heat-affected zone were significantly higher than the values of the temperature change away from the weld area. Therefore, the discrete model with mapped mesh was used to ensure the relative balance between the computer resource economy and the numerical simulation accuracy in order to ensure the convergence of the numerical simulation results [21,22]. In this study, hexahedral elements were used for meshing. The model had 27,830 mesh elements and 34,978 nodes, as shown in Figure 4. “A” represented the mesh structure of a part of the weld area.

### 2.4. Welding Heat Source Model

The numerical simulation of welding has conventionally been carried out by simulating the heat transfer in the weld using specific mathematical models. Significant stress concentration and deformation of the weld occurred due to the drastic increase in the temperature of the weld and the heat-affected zone. Therefore, the selection of a suitable heat source model was an important way to improve the accuracy of the numerical simulation. In this work, the double ellipsoidal heat source model invented by Goldak was used to represent the welding heat source, taking into account the phenomenon of arc penetration and temperature gradient distributions. The heat flux distribution resulting from a double ellipsoidal heat source was simulated for two front and back ellipsoids. The equations q_f_(x, y, z) and q_r_(x, y, z) were used to describe the front and rear power densities, respectively. The f_f_ and f_r_ were assumed to be the heat input in the front and rear parts of the ellipsoid, respectively, and f_f_ + f_r_ = 2. The equations of the double ellipsoidal heat source are as follows [23]:

(1) $$q_{f}(x,y,z)=\frac{6\sqrt{3}f_{f}E}{\pi a_{f}bc\sqrt{\pi }}exp(-3\frac{x^{2}}{a_{f}{}^{2}})(-3\frac{y^{2}}{b^{2}})(-3\frac{z^{2}}{c^{2}}),$$

(2) $$q_{r}(x,y,z)=\frac{6\sqrt{3}f_{r}E}{\pi a_{r}bc\sqrt{\pi }}exp(-3\frac{x^{2}}{a_{r}{}^{2}})(-3\frac{y^{2}}{b^{2}})(-3\frac{z^{2}}{c^{2}}),$$

(3) $$f_{f}=\frac{2a_{f}}{a_{f}+a_{r}},$$

(4) $$f_{r}=\frac{2a_{r}}{a_{f}+a_{r}}$$

and

**Figure 5:**
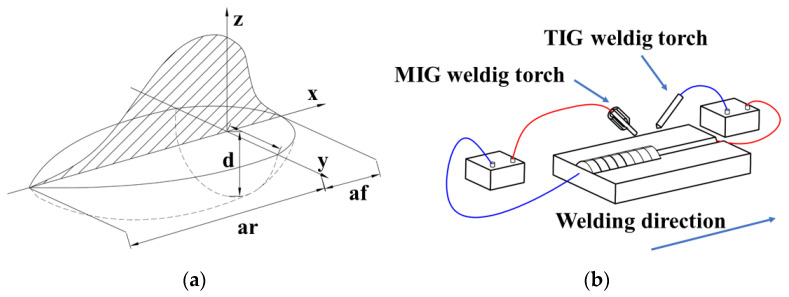
(**a**) Schematic double ellipsoid heat source model; (**b**) Schematic diagram of TIG-MIG hybrid welding.

(5) E=IU/v,

where I, U, v are the current, the voltage and the welding speed, respectively. The characteristic parameters of the double ellipsoidal heat source are labelled a, b and c. Figure 5a shows the schematic diagram of the double ellipsoidal heat source model.

The schematic diagram of the TIG-MIG hybrid welding process is shown in Figure 5b. The MIG welding process uses a reverse DC connection, which means that the weld is connected to the negative electrode and the wire to the positive electrode. In contrast to this, the TIG welding process uses a DC direct connection to suppress welding spatter and also to improve productivity [24].

### 2.5. Welding Thermal Cycle

T_8/5_ was the cooling time in the temperature range of 800 to 500 °C during the welding process. For the welding process, t_8/5_ was an important indicator of the mechanical properties of the welded joint, in addition to the effect of the welding heat input. It reflected the phase transition characteristics and mechanical properties of the weld and the heat-affected zone [25,26]. The cooling rate of the TIG-MIG hybrid welding simulation was discussed in this paper. The following equations are used to perform engineering calculations for the t_8/5_ value [27]:

(6) $$Q_{V}=m\cdot \eta \cdot E$$

and

(7) $$h_{c}=\sqrt{\frac{Q_{v}}{c\rho (T_{C}-T_{0})}},$$

where m is the form factor for heat dissipation, η is the thermal efficiency of the welding process, c is the specific heat, ρ is the density and T_0_ is the initial welding temperature. This value of T_C_ is typically chosen as 650 °C when calculating t_8/5_.

According to Rosenthal’s theory of heat transfer in welding, the t_8/5_ equations for thin plate welding (2D) and thick plate welding (3D) are:

(8) $$t_{8/5}^{2D}=\frac{Q_{V}{}^{2}}{4\pi c\rho \lambda h^{2}}\left[{\left(\frac{1}{500-T_{0}}\right)}^{2}-{\left(\frac{1}{800-T_{0}}\right)}^{2}\right]$$

and

(9) $$t_{8/5}^{3D}=\frac{Q_{V}}{2\pi \lambda }\left(\frac{1}{500-T_{0}}-\frac{1}{800-T_{0}}\right).$$

## 3. Results and Discussion

### 3.1. Comparison of TIG-MIG Hybrid Welding and Conventional MIG Welding Simulation

#### 3.1.1. Comparison of Two-Dimensional Graphs of Welding Simulation Temperature

**Figure 6:**
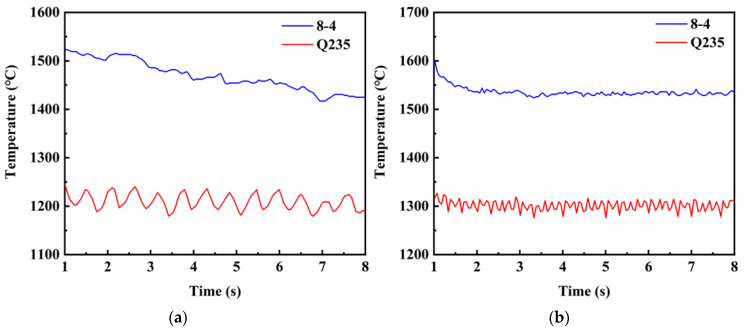
Comparison of the maximum temperature with different processes for weldments: (**a**) MIG welding; (**b**) TIG-MIG hybrid welding.

According to the research of Chen [28], the welding speed of the TIG-MIG hybrid welding could reach at least 1.5 m/min, and the weld was free from undercutting defects. In this paper, by controlling the same heat input for MIG welding and TIG-MIG hybrid welding at a high speed of 1.5 m/min, the first set of simulations was presented for comparison. Figure 6 shows the maximum temperature variation trends during welding using conventional MIG welding and TIG-MIG hybrid welding. It could be seen that, whether the base material was Q235 or the weld material was ZChSnSb8Cu4, the temperature variation of the conventional MIG welding was higher than that of the TIG-MIG hybrid welding. The temperature gradient of the hybrid welding was lower than that of the conventional MIG welding due to the preheating input and the lower heat loss to the weld provided by the TIG arc.

#### 3.1.2. Comparison of von Mises Equivalent Stress

**Figure 7:**
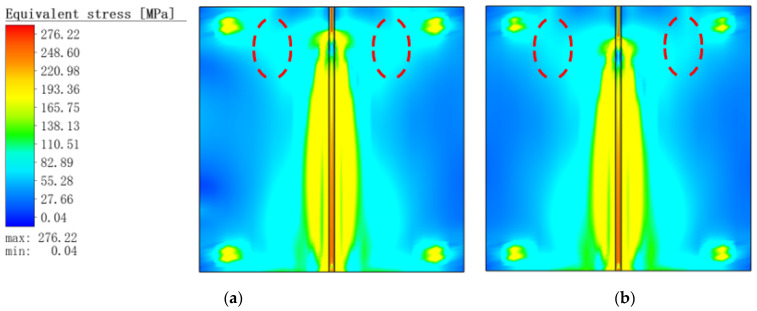
von Mises equivalent stress distribution at 7 s: (**a**) MIG welding; (**b**) TIG-MIG hybrid welding.

Figure 7 shows the von Mises equivalent stress distribution for the two operating conditions. It could be observed that the temperature increased rapidly, which led to the results that the internal weld and the heat-affected zone generated too much thermal welding stress during the welding process. Furthermore, at the 7 s time point, the von Mises equivalent stress values of the MIG welding were higher than those of the hybrid welding by 20~30 MPa at the connection of the heat-affected zone and the clamped area (red dotted area in the Figure 7). 

### 3.2. Distance between Electrodes for TIG-MIG Hybrid Welding

The welding heat input, the distance between the electrodes and the relative positions of the TIG and MIG torches were the main influencing factors in TIG-MIG hybrid welding.

In this section, the effect of the distance between the electrodes of TIG-MIG hybrid welding on the simulation results of the temperature field and welding thermal efficiency of the welding of layered bimetallic ZChSnSb8Cu4/steel was investigated. According to the experimental results of Lou [29], the electrode distance should be controlled between 3 mm and 10 mm when the TIG welding current was lower than the MIG welding current. In this paper, three electrode spacings of 6, 8 and 10 mm were set for this group of layer bimetallic ZChSnSb8Cu4/steel simulations.

**Figure 8:**
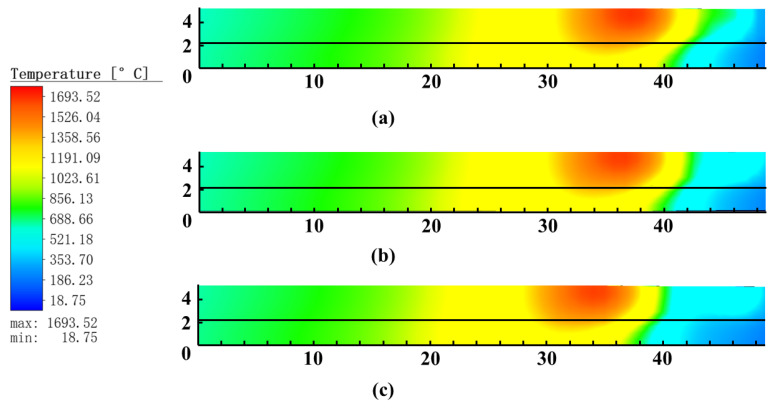
Temperature fields of layered bimetallic ZChSnSb8Cu4/steel at different distance between electrodes: (**a**) 6 mm; (**b**) 8 mm; (**c**) 10 mm.

**Figure 9:**
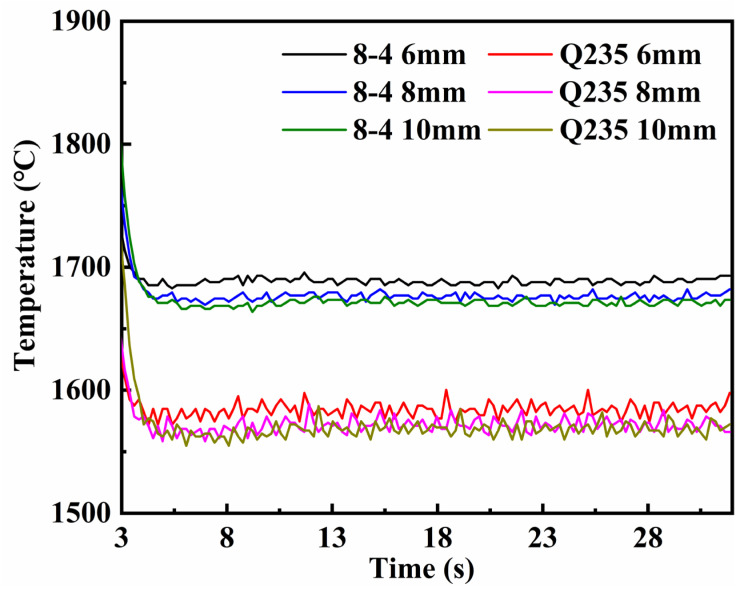
The relationship between the maximum temperature and time under different distance between electrodes.

Figure 8 shows the temperature distribution diagram of the cross section in the thickness direction of the weld during welding. It was found that the liquid layer at the front of the molten pool became progressively thinner as the distance between the electrodes increased, and the amount of high-temperature liquid Babbitt alloy decreased. The increasing distance between electrodes reduced the effect of the arc force, resulting in a reduction in the impact momentum and speed of movement of the molten droplets from the front heat source. According to the Bernoulli Effect, the reduction in the speed of movement of the molten droplets leads to an increase in the pressure on the surface of the molten bath and an increase in the reaction force towards the end of the molten bath. This increased the flow rate of high temperature liquid Babbitt alloy to the end of the pool, resulting in an increasingly large area of low temperature between the two arcs. Figure 9 also shows that the welding heat loss gradually increases as the distance between the electrodes increases.

From the results of the weld section temperature distribution chart in Figure 8, it could be seen that the temperature of the weld fusion zone at 6 mm pitch has exceeded the liquid phase line of the base material Q235 plate at 1517 °C. All of the base material in the fusion zone was in the form of liquid metal, which can melt relatively well with the liquid Babbitt alloy. When the distance between the electrodes was increased to 8 or 10 mm, the temperature of the Q235 in the heat-affected zone, which had reached the solid and liquid phase points of Q235, was significantly reduced. At this time, the weld fusion zone reached the solid-liquid coexistence state and could not be completely melted and combined with the liquid Babbitt alloy, which could have led to unfused weld defects. As a result of this series of simulations, it was observed that the best way to prepare the layered bimetallic ZChSnSb8Cu4/steel composite sheet was to control the distance between the electrodes at 6 mm.

**Figure 10:**
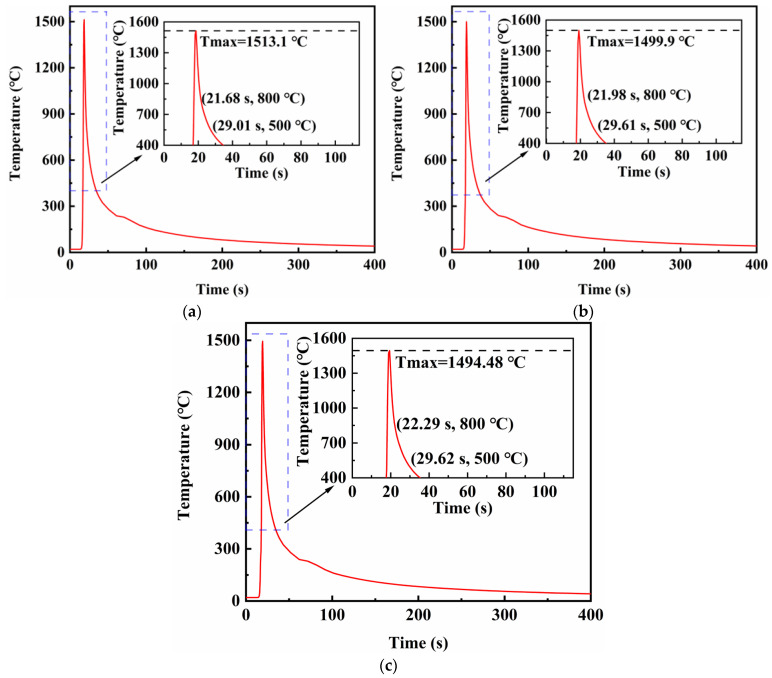
Thermal cycle curves at the weld fusion line when the distance between electrodes is: (**a**) 6 mm; (**b**) 8 mm; (**c**) 10 mm.

Figure 10 shows the thermal cycle curves for the center point of the fusion line in the middle of the weld under different electrode spacings. Welds at this point formed by spacings represented in Figure 10a–c reached their peak temperatures at 18.66, 19.09 and 19.37 s, respectively, and t_8/5_ values were 7.33, 7.63 and 7.33 s, respectively. Since the differences between the t_8/5_ values at the center of the weld and the fusion zone were very small, the thermophysical parameters such as specific heat capacity, thermal conductivity and density could be determined from the temperature at the center of the weld. Therefore, the actual thermal efficiencies were calculated as 0.672, 0.669 and 0.639, respectively. The results showed that as the distance between electrodes increased, the thermal efficiency of the welding gradually decreased and the heat loss increased. The blue dotted frame represents the part of the curve that needs to be magnified.

### 3.3. TIG-MIG Hybrid Welding Preheat Heat Source Current

In this group of simulations, the current, voltage and other parameters of the main heat source’s welding were consistent. The pre-heat source TIG welding current was gradually increased from 60 A to 80 and 100 A, to analyze the total deformation and stress changes in each weldment.

#### 3.3.1. Analysis the Total Deformation of Weldments

**Figure 11:**
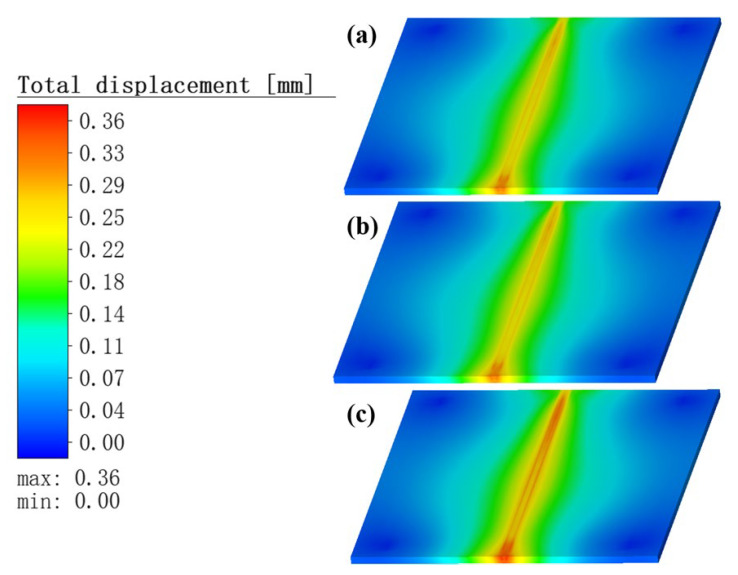
Distribution of the total deformation at the completion of welding. Current of TIG welding is: (**a**) 60 A; (**b**) 80 A; (**c**) 100 A.

**Figure 12:**
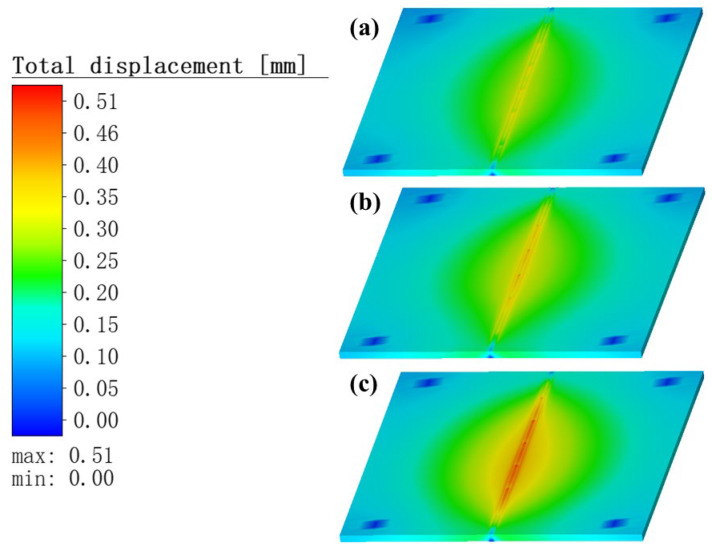
Distribution of the total deformation at the completion of cooling. Current of TIG welding is: (**a**) 60 A; (**b**) 80 A; (**c**) 100 A.

Figure 11 and Figure 12 show the distribution of the total deformation of the weldments at welding completion and at cooling completion, respectively. The results show that, as the TIG welding current gradually increased, the droplets of molten metal ejected from the TIG torch increased their speed of movement. Meanwhile, the thickness of the Babbitt alloy liquid channel in the middle part of the molten pool slowly decreased, and collected faster at the end of the molten pool. This caused the Babbitt alloy liquid layer in the middle of the molten pool to become progressively thinner and easier to solidify, which eventually prevented the molten droplets collected at the end of the molten pool from returning, and therefore tended to generate a hump phenomenon in the weld area. In contrast, in the layered bimetallic ZChSnSb8Cu4/steel simulation, when the welding process was completed and when the cooling process was completed, increased current in the preheating heat source led to increased heat input, resulting in a slowly increasing temperature gradient of the specimen and a small increase in the thermal stress generated. The total deformation during the welding process increased with the current used in the TIG welding.

**Figure 13:**
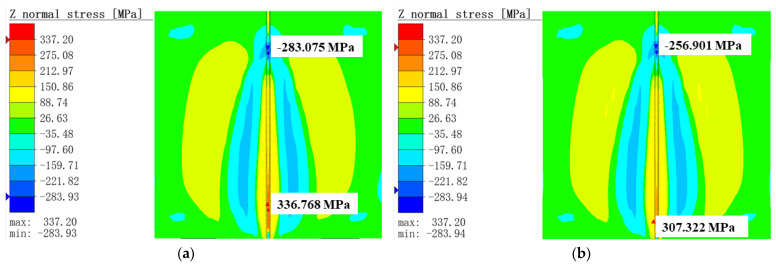

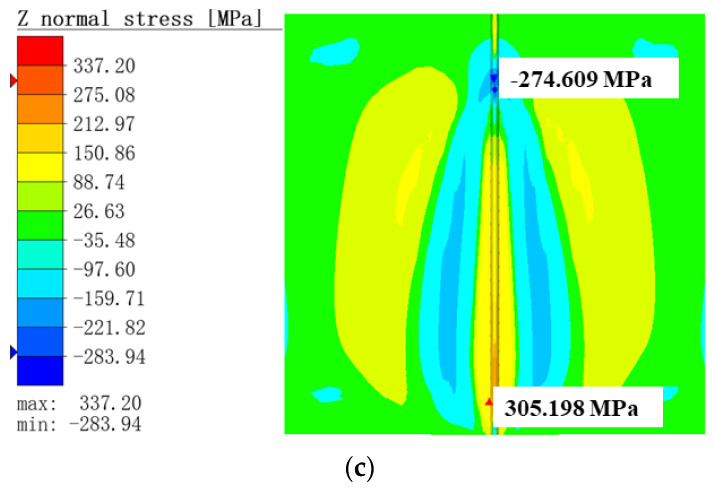
Stress distribution in weld direction at 27 S welding. Current of TIG welding is: (**a**) 60 A; (**b**) 80 A; (**c**) 100 A.

During the simulation of layered bimetallic ZChSnSb8Cu4/steel welding, the actual temperature of the weld area was higher compared to both sides, while the temperature of the rest of the non-welded area decreased as the welding time or the length of the weld increased with the movement of the welding heat source. Welding tensile and compressive stresses were generated by temperature changes during welding, resulting in changes in the amount of expansion and contraction per unit length of the weld. The bimetallic ZChSnSb8Cu4/steel plate was designed to maintain structural stability and limit the expansion and contraction of the weld. Figure 13 shows the stress distribution in the weld direction for the three TIG welding currents at 27 s. The weld material near the heat source expanded due to the sharp increase in temperature, and the compressive stress was generated by the base material exerting an extrusion effect on the weld. Compressive plastic deformation occurred when the compressive stress exceeded the yield stress of the base material of the weld, resulting in a more stable, flat plate structure. Conversely, the other areas in the weld would be stretched due to the shrinkage of the weld, which meant that weld tensile stresses were generated. Figure 13 also shows that the peak tensile and compressive stresses were greatest at a TIG welding current of 60 A. As the TIG welding current increased, the peak tensile stress gradually decreased to 305.2 MPa, and the peak compressive stress decreased and then increased to 274.61 MPa.

#### 3.3.2. Analysis Residual Stress

**Figure 14:**
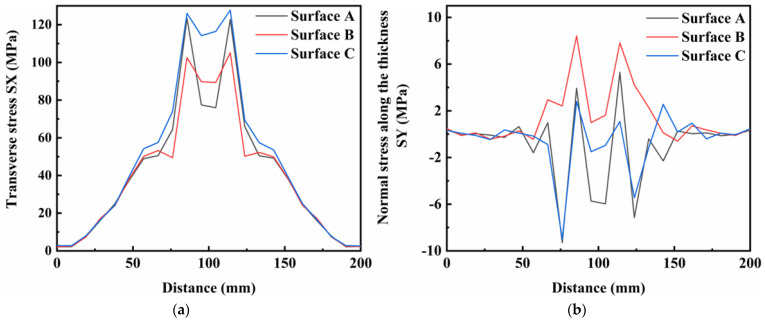

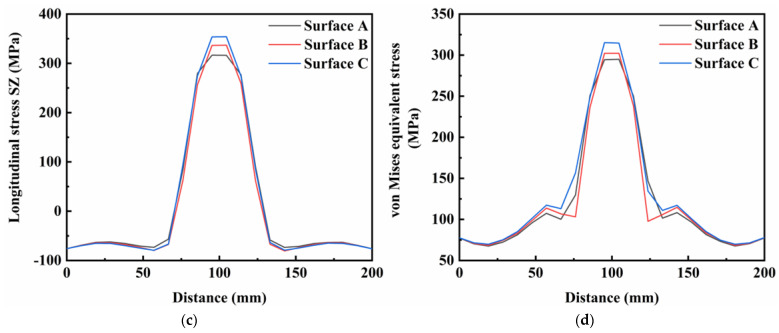
Residual stress in weldment thickness direction under TIG welding current of 60 A: (**a**) Transverse stress; (**b**) Normal stress along the thickness; (**c**) Longitudinal stress; (**d**) von Mises equivalent stress.

Figure 14a–c shows the normal stress components at different weldment thicknesses at a preheating heat source current of 60 A. The weld was divided into three surfaces for analysis from top to bottom and these were designated as surface A (top of the specimen), surface B (middle of the specimen) and surface C (bottom of the specimen), respectively. It was found that the distribution characteristics of the three surfaces were similar for the transverse stresses, and that the whole of the plate was in a state of tensile stress. At the bottom of the weld, the transverse tensile stresses in the weld area were particularly pronounced. Furthermore, the residual stresses in the thickness direction of the weldment were basically similar in the three surfaces of A, B and C. However, the compressive stress values in the surface A and B of the weldment were higher in the area close to the weld seam. For longitudinal stresses, all three surfaces exhibited high tensile stress values in the region close to the weld, while the region away from the weld seam was in a state of compressive stress. The longitudinal tensile stress values at the surface C of the weld area were large, and the structural stability of this area required greater focus during the experiments. Figure 14d shows the distribution of von Mises equivalent stresses at the surfaces A, B and C. In general, the distribution characteristics were similar in different areas of the weldment thickness direction, but the peak von Mises equivalent stress increased from the top to the bottom of the weldment.

**Figure 15:**
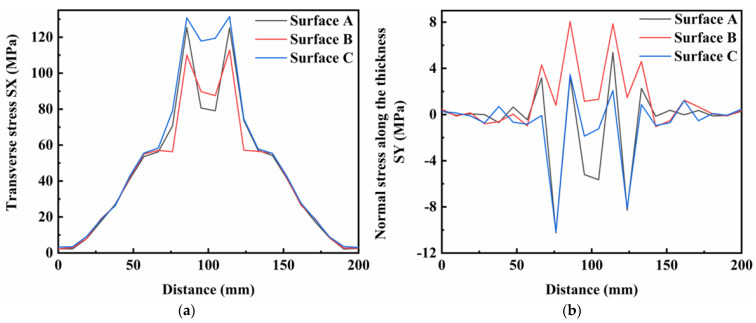

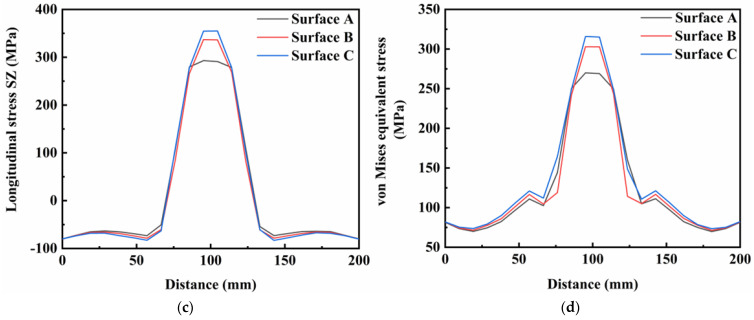
Residual stress in weldment thickness direction under TIG welding current of 80 A: (**a**) Transverse stress; (**b**) Normal stress along the thickness; (**c**) Longitudinal stress; (**d**) von Mises equivalent stress.

**Figure 16:**
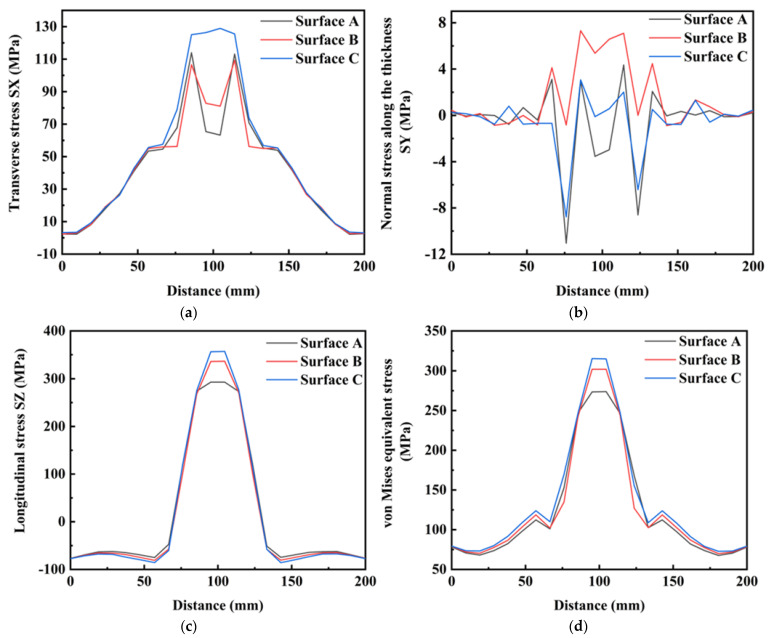
Residual stress in weldment thickness direction under TIG welding current of 100 A: (**a**) Transverse stress; (**b**) Normal stress along the thickness; (**c**) Longitudinal stress; (**d**) von Mises equivalent stress.

Figure 14, Figure 15 and Figure 16 show the residual stress distributions at different positions in the weldment thickness direction for TIG currents of 60, 80 and 100 A, respectively. The results showed that the residual stress distributions remained basically the same as the TIG welding current increased, and the peak value of stress was generally increased. However, when the TIG current was increased to 100 A, there was a significant increase in the transverse stress in the weld area at the bottom of the weldment, while the transverse stress in the weld area at the top of the weldment decreased by about 10 MPa. Although the overall stress values of the residual stresses in the thickness direction were relatively low, the stress value in the middle of the weld area increased by about 5 MPa as the current increased. The stress value in the weld area at the top of the weldment increased by 2 MPa, and the stress value at the bottom of the weldment increased, but was less than in the other two areas. The increased TIG current resulted impacted the longitudinal stresses and von Mises equivalent stresses, with an increase in the peak residual stress and in the three surfaces in the thickness direction of the weldment, while the stress value in the surface A of the weldment decreased by approximately 20 MPa.

**Figure 17:**
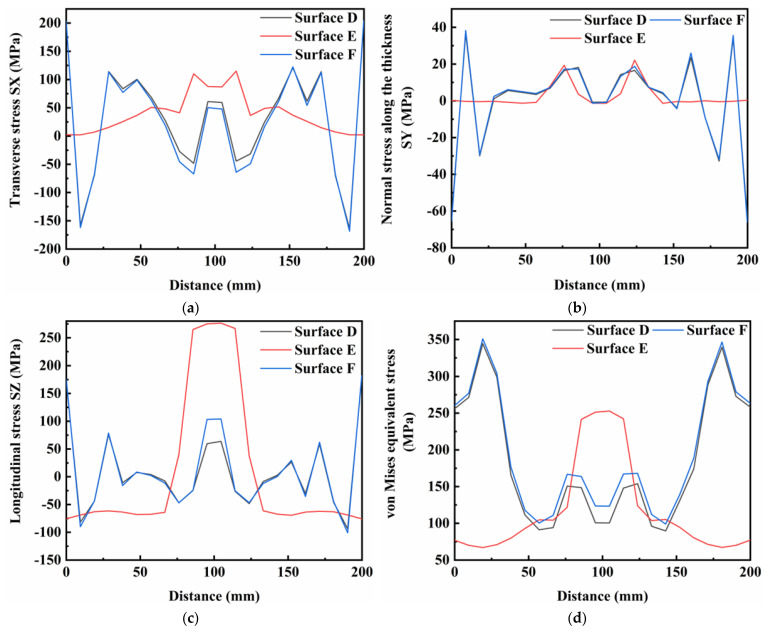
Residual stress on top surface of weldment under TIG welding current of 60 A: (**a**) Transverse stress; (**b**) Normal stress along the thickness; (**c**) Longitudinal stress; (**d**) von Mises equivalent stress.

Figure 17a–d show the distributions of residual stresses on the top surface of the weldment along the vertical welding direction for the preheating heat source current of 60 A. The area clamped by the fixed geometric points at the first and last ends of the weldment were designated as surfaces D and F, and the middle of the weldment was designated as surface E. Similar trends of the stress components and the von Mises equivalent stress were noted between the surfaces D and F, but these differed significantly from that of surface E. Areas clamped by the fixed geometry points were subjected to both transverse and longitudinal stresses, and the area away from the weld seam was subjected to higher tensile stresses. In surface E, transverse stresses were subjected to tensile stresses, and longitudinal stresses were subjected to tensile stresses in the area close to the weld. However, the stress on the surface of the workpiece along the thickness of the plate was much less than the others. The von Mises equivalent stresses, in both the clamped area and the middle of the weldment, were affected by tensile stresses.

**Figure 18:**
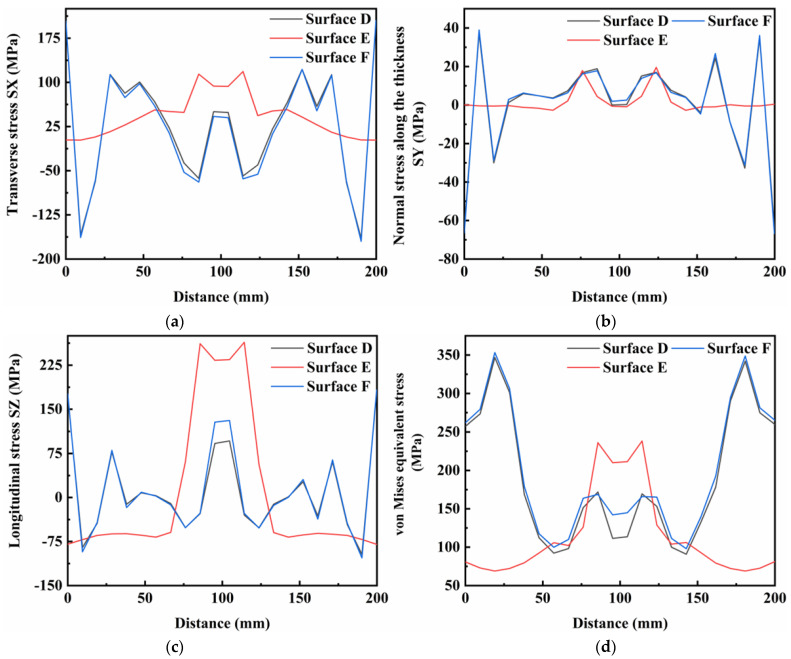
Residual stress on top surface of weldment under TIG welding current of 80 A: (**a**) Transverse stress; (**b**) Normal stress along the thickness; (**c**) Longitudinal stress; (**d**) von Mises equivalent stress.

**Figure 19:**
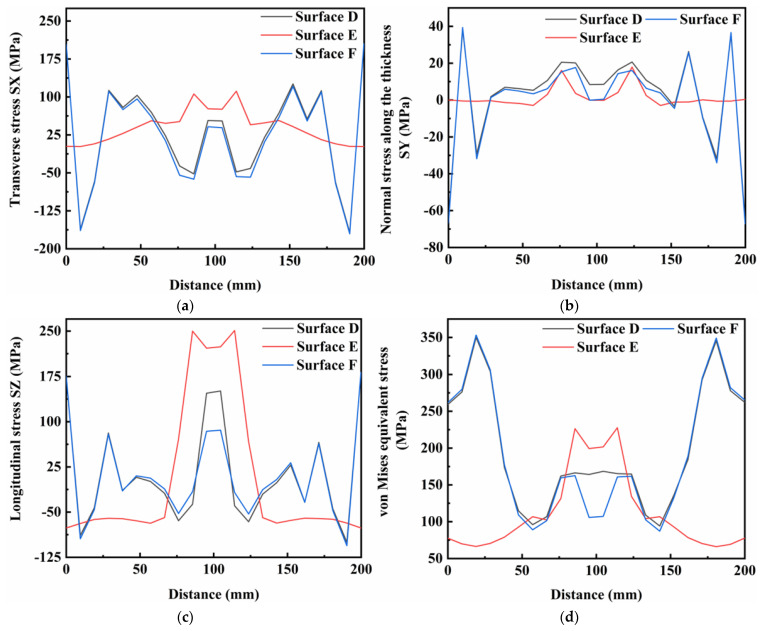
Residual stress on top surface of weldment under TIG welding current of 100 A: (**a**) Transverse stress; (**b**) Normal stress along the thickness; (**c**) Longitudinal stress; (**d**) von Mises equivalent stress.

Figure 17, Figure 18 and Figure 19 show the distributions of residual stresses on the top surface of the weld along the vertical weld direction at TIG currents of 60, 80 and 100 A, respectively. As the TIG current was gradually increased, the stress distributions in the transverse stress and thickness directions showed the similar characteristics. From the distributions of longitudinal stress and von Mises equivalent stress, the stress values in the surface E of the weld area were dramatically reduced by approximately 50 MPa, and the stress values in the surfaces D and F of the weld decreased by 20 MPa. However, the stress values in the surfaces D and F of the weld area increased by 50 MPa.

The above figures illustrate the physical parameters of the material and the trend in residual stress with different welding directions under certain conditions. A key finding was that there was no positive proportional relationship between the current and the residual stress distribution in the welding thickness direction or in the top surface of the weld. However, the results under other certain conditions were difficult to assume due to the properties of materials, such as melting point of the material and surface tension of molten pool. Therefore, regression analysis, mathematical models and approximation reliability coefficients are not given here.

**Figure 20:**
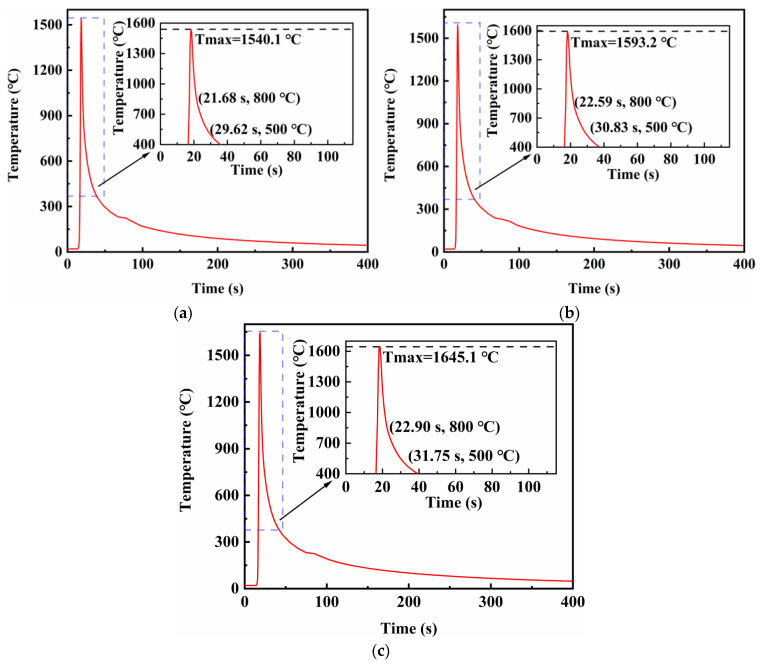
Thermal cycle curves at the weld fusion line when the current of TIG welding is: (**a**) 60 A; (**b**) 80 A; (**c**) 100 A.

According to Figure 20 and the dynamic graph in the supplementary files, the fusion lines in the middle of the weldment reached the peak temperature at 18.66 s for all three TIG welding currents, 60, 80 and 100 A, and the t_8/5_ values were 7.94, 8.24 and 8.85 s, respectively. The actual thermal efficiencies were calculated as 0.678, 0.668, and 0.669, respectively. Consequently, the thermal efficiency of the whole simulation was the highest when the TIG welding current was 60 A. The blue dotted frame represents the part of the curve that needs to be magnified.

## 4. Conclusions

The TIG-MIG hybrid welding process and numerical simulation were proposed to solve the welding spatter problem of layered bimetallic ZChSnSb8Cu4/steel in welding. The obtained results are summarized as:

- Based on the simulation results, TIG-MIG hybrid welding showed superiority in heat loss and stress concentration. At the connection of the heat-affected zone and the clamped area, the von Mises equivalent stress values of the MIG welding were higher than those of the hybrid welding by 20~30 MPa.
- When the distance between the electrodes of TIG-MIG hybrid welding was 6 mm, heat loss was minimized and the weld seam was completely fused. Increasing the distance between the electrodes led to an increase of the low temperature region, and the welding thermal efficiency gradually decreased to 0.639.
- As the current of the TIG torch increased, the total deformation in the weld area became more significant due to the increased extrusion of the substrate on the weld, caused by the temperature change. The increase in current of the TIG torch also caused significant changes of stress values in the weld and clamping areas of the weldment. When the TIG current was 100 A, the equivalent stress in the weld area increased by about 50 MPa.

## Figures and Tables

**Table 1:** Physical thermodynamic parameters of Q235 steel plate.

Property	Value	Unit
Environment temperature	20	°C
Thermal conductivity coefficient	0.05	10^3^ W·m^−1^·°C^−1^
Heat transfer coefficient	1.0	10^2^ W·m^−2^·°C^−1^
Density	7.8	10^3^ kg·m^−3^
Specific heat	460	J·g^−1^·°C^−1^

**Table 2:** Main elements of ZChSnSb8Cu4 welding wire (mass fraction, %).

Sb	Cu	Pb	As	Sn
8.0	4.0	0.35	0.1	balance

**Table 3:** Main elements of Q235 steel (mass fraction, %).

C	Si	Mn	P	S	Fe
0.16	0.15	0.37	0.014	0.010	balance

## Data Availability

Not applicable.
